# Supplementary material for: Photobiomodulation reduces hippocampal apoptotic cell death and produces a Raman spectroscopic “signature”
Source: PLoS One. 2022 Mar 3;17(3):e0264533. doi: 10.1371/journal.pone.0264533 (PMC8893683; doi:10.1371/journal.pone.0264533)
Supplement: Attachment — Submitted filename: Response to Reviewers.docx [file pone.0264533.s001.docx]

Institute of Inflammation and Ageing

University of Birmingham

Edgbaston

Birmingham

B15 2TT

UK

3^rd^ February 2022

Dear Prof. Hamblin,

On behalf of all of our co-authors, I would like to extend our sincere thanks to you and the reviewers for the time and consideration of our article. We were greatly encouraged to read the positive responses from the reviewers on the scientific merit of our submitted work.

We have taken time to consider and act upon the suggestions for improvement offered to us by Reviewer 2, and we enclose an updated manuscript as requested which reflect the changes. The authors agree that the updated manuscript, with the changes described below, represents a considerable improvement to the original work, and we offer our further thanks for such comments for improvement.

The response to each suggestion is enclosed below in bold type face, with comments above in italics, and numbered for ease of reference.

*1. On line 30, for the sentence, you can cite recently published papers (e.g., PMID: 29164625; PMID: 29327206).*

**Response: These citations have been added**

*2. The English writing quality of the manuscript should be improved.*

**Response: The document has been reviewed in its entirety, with minor changes made to grammar, phrasing and syntax which we hope the reviewers will agree has made a considerable improvement to the writing quality, without affecting the scientific content.**

*3. On line 76, PBM parameters such as irradiance, fluence, the dose should be reported as follow, respectively, mW/cm2, J/cm2, and J. The use of "irradiance, fluence, the dose" should be consistent throughout the manuscript.*

**Response: This sentence has been updated, and this nomenclature is reflected where mentioned and throughout the manuscript.**

*4. On line 104, Irradiance refers to "Power density" not "Photon density".*

**Response: We are in agreement with the reviewer that irradiance is not referring to “photon density”. The term “irradiance” has now been left, without further qualification, given clear establishment of terms relating to PBM parameters as suggested in point 3.**

*5. On the paragraph starting from line 89, the functional wavelength spectrum of the Raman spectroscopy could be mentioned. In other words, this technique is sensitive to what particular range of the spectrum (e.g, visible, near-infrared, mid-infrared, far-infrared, etc)?*

**Response: We have added a further sentence which clarifies the typical wavelengths used in Raman spectroscopy for biological samples, and the respective wavenumbers which are detected and typically represent spectra from biological molecules. We feel this is an adequate though succinct summary which addresses the point without over-elaboration on RS to a non-specialist readership.**

*6. On line 153, "irradiance (power)" should be changed to "irradiance (power density)".*

**Response: As discussed in the response to point 4, we have changed this to: “irradiance”**

*7. PBM parameters should be completely reported; Output power, fluence, beam diameter and area, beam profile (Gaussian or Hot top), the distance of the LED probe from the dishes, etc.*

**Response: We have added further detail as suggested. Irradiance values given have been measured at the level of the tissue plane, and this has been made clear in the revised manuscript and is now accompanied by respective fluence values for 1 min treatment time. With the images in figure 2, and with the well diameter, beam diameter and fluence values now given, we feel this is a complete reporting of the salient parameters (as outlined in our group’s previous work on the topic (PMID: 29694800).**

*8. In the Discussion section, some of the previous in vitro studies on positive neuroprotective effects of 2.5 J/cm2 red light on neuronal cell culture can be discussed (see PMID: 33935090).*

**Response: We thank the reviewer for their suggestion and have included a short discussion with reference to these articles.**

We have also conformed to the other journal requirements advised in the correspondence. Formatting has been adjusted to reflect the PLOS ONE guidelines. Reference changes were as follows: references 9, 10, and 38-41 have been added.

In response to the request for an amended statement of competing interests, we include the below which has been updated within the attached revised manuscript:

The development of this novel concept has resulted in a patent pending application from our group relating to the invasive delivery of PBM, together with the use of temporarily implanted apparatus to establish an optimal dose feedback loop via an optical spectroscopic brain interface (UK Patent Application No 2006201.4). There are no other competing interests to declare, including those relating to employment, consultancy, other patents or products in development. The authors confirm that this does not alter our adherence to all PLOS ONE policies on sharing data and materials.

Yours sincerely

Mr David Davies
